# A Spatio-Temporal Approach to Individual Mobility Modeling in On-Device Cognitive Computing Platforms

**DOI:** 10.3390/s19183949

**Published:** 2019-09-12

**Authors:** Rafael Pérez-Torres, César Torres-Huitzil, Hiram Galeana-Zapién

**Affiliations:** 1 School of Computer Science & Information Technology, University College Cork, T12 YN60 Cork, Ireland; 2 Tecnologico de Monterrey, School of Engineering and Sciences, Campus Puebla, Puebla C.P. 72453, Mexico; torresc@tec.mx; 3 Cinvestav Tamaulipas, Ciudad Victoria C.P. 87130, Mexico; hgaleana@tamps.cinvestav.mx

**Keywords:** human mobility, trajectory, POI, cognitive computing, smartphone

## Abstract

The increased availability of GPS-enabled devices makes possible to collect location data for mining purposes and to develop mobility-based services (MBS). For most of the MBSs, determining interesting locations and frequent Points of Interest (POIs) is of paramount importance to study the semantic of places visited by an individual and the mobility patterns as a spatio-temporal phenomenon. In this paper, we propose a novel approach that uses mobility-based services for on-device and individual-centered mobility understanding. Unlike existing approaches that use crowd data for cloud-assisted POI extraction, the proposed solution autonomously detects POIs and mobility events to incrementally construct a cognitive map (spatio-temporal model) of individual mobility suitable to constrained mobile platforms. In particular, we focus on detecting POIs and enter-exits events as the key to derive statistical properties for characterizing the dynamics of an individual’s mobility. We show that the proposed spatio-temporal map effectively extracts core features from the user-POI interaction that are relevant for analytics such as mobility prediction. We also demonstrate how the obtained spatio-temporal model can be exploited to assess the relevance of daily mobility routines. This novel cognitive and on-line mobility modeling contributes toward the distributed intelligence of IoT connected devices without strongly compromising energy.

## 1. Introduction

The widespread use of Global Positioning System (GPS)-enabled mobile devices has enabled the unobtrusive collection of myriads of location data for mobility analysis. As an example, mobility mining at community level [1] allows to understand the dynamics of populations, the spatial distributions, and the categorization of mobility patterns of users in different communities [2]. Furthermore, the development of human mobility-based services (MBS) has produced applications that adapt their behavior according to the user’s position and past mobility traces [3]. These applications include recommender systems [4,5], individual’s mobility profiling [5], mobility prediction using Hidden Markov Models (HMM) [2,6,7], among others. Notably, the information generated by MBSs is of high relevance for large-scale developments, such as urban planning, traffic management, and more.

Mobility analytics commonly relies on a Mobile Cloud Computing (MCC) architecture on which the mobile device continuously acquires and transmits its location to a cloud-based system for off-line processing [8]. Nevertheless, the transition to Internet of Things (IoT) technologies demands new requirements, such as autonomous operation, self-awareness, and fully distributed intelligence of devices [9], which are hard to effectively achieve by relying exclusively on MCC. Additionally, MBSs in IoT must rely on simple yet efficient data processing techniques to achieve extended and continuous localization monitoring for accurate mobility understanding, also known as power minimized services [10]. Such techniques should also consider that location data streams might be collected asynchronously, i.e., without a defined sampling frequency.

For IoT MBSs, on-device decision making enables on-line features with advantages over the MCC approach [11]. For instance, on-line location analysis would provide nearly real time reactions at user-specific spatio-temporal scales [5], a highly desirable feature in navigational services, assisted driving, etc. Also, on-device solutions reduce the cloud dependency and issues related to the time-varying wireless channel conditions, leading possibly to disconnections and/or uplink capacity bottlenecks. Furthermore, as location data transmissions to the cloud are mostly avoided, the on-device approach helps to deploy solutions with consistent performance regardless of the locally available infrastructure (3G, 4G); legal issues when offloading data to clouds in different countries are also avoided. Attending to these issues, we strongly believe that an on-device approach gives a more proactive role to connected devices in large-scale IoT systems. Given their large number, it is very plausible that many devices will be idle at every instant [12]. Thus, a distributed and on-device approach will contribute to alleviate backhaul links bottlenecks, and to address MCC issues like device energy consumption, latency, and privacy reinforcement [9,12].

In this context, in our previous work [13] we proposed a novel on-device cognitive sensing framework for IoT devices, on which we validated that cognitive features could be of use to characterize mobility from POIs and basic mobility events. Unlike existing approaches for on-device location tracking, we aim to enable the device with self-mobility understanding and contribute toward the distributed intelligence demanded by the future Cognitive IoT [14].

Existing work on mobility modeling focuses on two kinds of approaches: spatio-temporal modeling and spatial movement modeling. The former aims to discover the prominent daily temporal habits as well as predicting future individual activities, meanwhile the later facilitates next-place and residence time prediction and the identification of departures from mobility routines [2]. While probabilistic models (HMM) and traditional data mining algorithms (spatial clustering) work under the assumption that data is stored and always accessible, it becomes challenging to produce similar results on-the-fly. Thus, there is the need for solutions that model and extract mobility events from the GPS stream along with the plausibility to be implemented on device. In this article, we demonstrate how user’s mobility can be automatically captured in a comprehensive representation, a cognitive map, as an expanded spatio-temporal model suitable for further on-device individual mobility analysis. It is worth noting that although the term cognitive map has been employed in other disciplines to indicate the representation that humans mentally create from the space we interact with, in this work cognitive map refers to the spatio-temporal model that is created by a Cognitive Dynamic System. The cognitive map is constructed from events, structurally related to each other in the context of space and time, raised from the interaction between the device and its environment (POIs) during a learning stage; then in a testing stage this information could be exploited for mobility mining.

Our main contributions are: (a) a user-tailored spatio-temporal representation of mobility (referred to as a cognitive map), which is constructed from long exposure to simple individual mobility events, and (b) an event-based implementation consisting in a continuous perception-action cycle that incrementally constructs and updates the cognitive map on GPS-enabled mobile devices. Our cognitive map reflects the observed mobility from individuals, characterizing their regularity or patterns (if any). We validate the spatio-temporal accuracy of our approach to construct the cognitive map using an annotated database. Also, we outline how this cognitive map could be further exploited for mobility estimation and prediction, provided some regularity, without incurring in excessive computational and energy overheads. This shows experimental evidence for both, the accurate construction of the map with respect of fine-grain location data, and its exploitation for mobility mining on constrained mobile devices.

The rest of this article is organized as follows. Section 2 summarizes the background of POIs, event-based systems, and the Cognitive Dynamic Systems (CDSs) framework. Section 3 describes the event-based representation of mobility and the spatio-temporal modeling to construct the cognitive map. Section 4 shows how the map can be further exploited for mobility prediction and place categorization, as well as its assessment based on spatio-temporal entropy. Experimental results are presented in Section 5, followed by a discussion in Section 6. Concluding remarks are presented in Section 7.

## 2. Background

Characterizing the statistical properties of individual trajectories helps to understand the dynamics of human mobility. Broadly speaking, we consider a trajectory as the displacements between POIs and pauses at them, and we argue that studying the distribution of these pauses and traveled distances helps to assess such dynamics [15,16]. Interestingly, people have a very small number of POIs that are daily visited, and a higher but still limited number of POIs that are less frequently visited [17]. As a result, the study and modeling of human mobility is feasible. We present a concise review of related approaches, some background of POIs, and event-based and cognitive frameworks. Herein after, we employ the symbols and notations in Table 1.

### 2.1. Related Approaches

In this study, we focus on the use of mobile phone data to understand human mobility from the interaction of people with POIs. Previous work on this illustrates a pattern of preferential returns to previously visited locations and explorations of new places as a general and universal feature.

Yuan, et al. [18] proposed a method for off-line GPS trajectory analysis to detect places and travel sequences. The method splits a trajectory in segments delimited by endpoints that represent the stop locations (POIs), so that most of the fixes are discarded in further computations. Endpoints are used to characterize a network of frequent paths between POIs, which are then employed to predict next user POI, provided the current trajectory is similar to existing ones. In [19], authors present a mechanism to produce a compressed graph of an individual’s mobility. Authors identify POIs from cellular ID records, filtering those to which a user has been connected to for longer than an hour. Then, two stay points would be connected with an edge if the user has moved between their corresponding cells; the weight if the edge is the average traversing time. The sequence of POIs is used to build a graph, which is then compressed for a lightweight representation of the user’s mobility using the Shrink method. Similarly, in [20] a mechanism to construct an individual-tailored mobility model is proposed. Using the off-line algorithm in [21], authors calculate POIs and group them by spatial similarity. The POIs are sorted by arrival time and then are connected to reflect the sequence of POIs visited during the day in a directed graph. The more connections between POIs, the larger the edge weight. Another example of POIs sequence construction is proposed in [22], on which POIs are also detected using the algorithm in [21], for being later grouped using a hierarchical clustering algorithm. Clusters are assigned a semantic meaning (gym, store, etc.) using OpenStreetMap and authors construct the trajectories corresponding to the sequence of visits to such places. The trajectories are clustered using language processing techniques, which assist the prediction of places when a new trajectory is provided.

Notice that the above mining methods are founded on the assumption that data instances are independent and sometimes expecting that data is sampled with a constant frequency. Ignoring that data is structurally related to each other, both in space and time, can lead to poor accuracy and interpretability. Moreover, it is desirable to find suitable algorithms to extract meaningful stay locations for further analysis, reducing the noise in the big data.

In [23], Kang et al. proposed a method suitable for mobile devices to calculate POIs in an on-line manner, based on spatio-temporal differences between individual location fixes. Such method queues fixes in a cluster as long as the traveled distance and elapsed time are within thresholds. Nevertheless, the distance evaluation and the queuing of fixes in a cluster requires the frequent recalculation of a cluster’s centroid, which can be an issue if many fixes are to be queued. Similarly, the connections between detected POIs are not investigated, and as a result such work merely acts as a yet efficient [24] POIs detection mechanism, very much alike the ideas we explored before in [25].

In this regard, our solution provides novel features for mobility analytics, as shown in Table 2. In first term, the solution is completely on-device, suppressing the need for sharing data with external entities, which improves energy efficiency and contributes to user’s privacy since data is always kept locally. Secondly, it goes beyond the scarcely existing on-device approaches for POIs detection, as it explores the temporal attributes of the connections between POIs and how they can be exploited for further tasks such as POIs prediction and categorization. Such information is encapsulated in a spatio-temporal model that is incrementally constructed and updated reflecting user’s mobility and associated events, using the concepts described in the following text.

### 2.2. Trajectory and Points of Interest

Herein, we consider a trajectory as a sequence of GPS fixes, $Trj=\{f_{m},f_{m+1},\ldots ,f_{n}\}$, and each fix, $f_{k}=\left(x,y,t\right)$, with *x*, *y*, *t* as its latitude, longitude, and timestamp, respectively. The sequence’s length is l=|Trj|=n−m+1, observed during the time interval $\left[f_{m}.t,f_{n}.t\right]$.

Since trajectories intrinsically include time, they provide different semantics depending on whether they are analyzed according to spatial or spatio-temporal proximity [26]. A POI refers to a trajectory Trj restricted within a geographical region with a spatial distance threshold $D_{max}$, where a user spent $T_{min}$ units of time:

(1) $$\begin{array}{c} f_{n}.t-f_{m}.t\geq T_{min} \end{array}\;\begin{array}{c} d_{hav}\left(f_{m},f_{i}\right)\leq D_{max},\forall m<i\leq n \end{array}$$

where $d_{hav}$ is the Haversine distance between two fixes.

A POI is defined as $POI=(\left\{x_{poi},y_{poi}\right\},t_{in},t_{out})$, where $\left\{x_{poi},y_{poi}\right\}$ is the POI’s centroid computed as:

(2) $$\begin{array}{c} x_{poi}=\frac{\sum_{i=m}^{n}f_{i}.x}{l} \end{array}\;\;\;\begin{array}{c} y_{poi}=\frac{\sum_{i=m}^{n}f_{i}.y}{l} \end{array}$$

and $t_{in}$, $t_{out}$ are the enter and exit times, respectively.

### 2.3. Event-Based Processing

Events are real-world occurrences that unfold over space and time, and that might involve a change in system’s state. Events can be atomic or complex. An atomic event is defined as $ev_{at}=ev\left(id,\left\{a_{1},\ldots ,a_{n}\right\},t\right)$, where id is its unique identifier, $a_{1},\ldots ,a_{n}$ are a set of *n* attributes that characterize it, and *t* is its occurrence timestamp. A complex event is defined as $ev_{comp}=ev\left(id,p,\left\{a_{1},a_{2},\ldots ,a_{n}\right\},t_{s},t_{e}\right)$, and it is composed from atomic events using a function *p*, with $t_{s}$ and $t_{e}$ as its starting and ending times, satisfying $t_{s}\leq t_{e}$. Instant-defined events are possible by specifying $t_{s}=t_{e}$.

An event stream $s_{ev}$ is an ordered event sequence, $s_{ev}=s_{ev}\left(ev_{1},ev_{2},\ldots ,ev_{n}\right)$, where $ev_{i}$ is atomic or complex. An event processing module consumes events and creates new (derived) ones using a set of *p* functions. The event processing module, event producers, and event consumers are inter-connected through push-style communication channels.

Notice that an atomic event, as the singlest piece of data that can be retrieved from sensors, can be associated with a single location fix, and a complex event can refer to an activity or behavior that is calculated from atomic events, for instance, a POI or the enter and exit from it. As it will be described later on, event-driven processing suits the process of complex activity recognition (in this case mobility), attending to the rich temporal dependencies between POIs and associated events [27].

### 2.4. Reference CDS Framework

**Figure 1:**
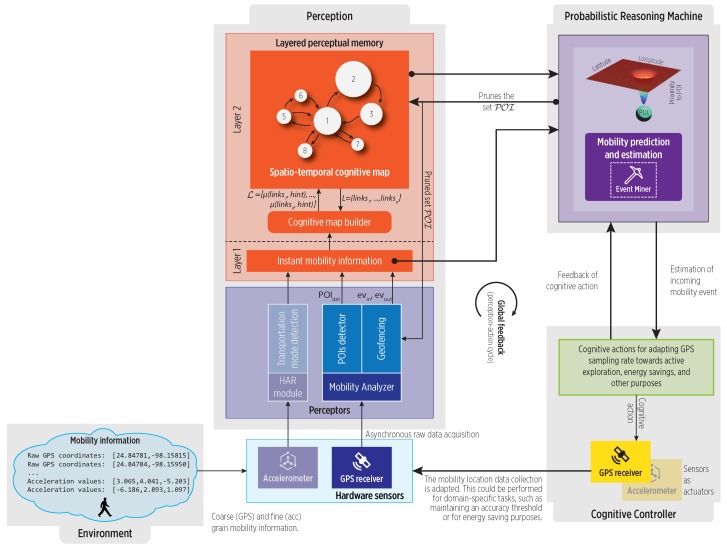
The architecture of the reference CDS for on-device mobility detection, learning and understanding (enhanced from [13]). the perception block constructs the cognitive map to summarize mobility for further exploitation and analysis in the PRM.

Herein, we briefly describe the reference on-device cognitive (CDS) framework proposed in [13] for POIs and mobility events detection. Our CDS, overall architecture shown in Figure 1, relies on a continuous perception-action cycle for events detection and mobility characterization in the cognitive map. The perceptors incrementally process asynchronous location data collected from the GPS receiver to detect new POIs and enter-exit events. We claim that long exposure to such events can help to incrementally summarize regular features of mobility in the form of a cognitive map, which would reside in the CDS perceptual memory. The map construction approach in this paper is only a possible way, yet simple and efficient, but other event-driven strategies could be employed as well.

The Probabilistic Reasoning Machine (PRM) could be able to exploit such cognitive map for knowledge extraction, mobility mining tasks like events predictions, and for generating cognitive actions. In the reference framework, cognitive actions are goal-oriented, for instance, in on-device MBSs adaptive GPS sampling can reduce energy consumption while performing an accurate perception. In this sense, a cognitive map can be considered as a basic component for adaptive behavior.

## 3. Spatio-Temporal Cognitive Map

We focus on creating a cognitive map of individuals mobility based on their interaction with POIs to profile daily routines, which can contribute to and infer needs and social interaction [8]. Although many factors cannot be fully accounted through opportunistic sensing [28], core spatio-temporal features of individual mobility could be captured using sensors data and machine learning methods to structure the physical environment.

### 3.1. Event-Based Mobility

Using the event-based paradigm (see Section 2), we provide an event-based view of an entity’s trajectory and its motion patterns as follows. First, each location fix is an atomic event, and a trajectory is a stream of fixes (i.e., a sequence of events). Second, a POI is a complex event calculated from a trajectory by a function *p* based on the concepts of Section 2.2 (i.e., a sequence of events that fulfills time and spatial constraints).

**Figure 2:**
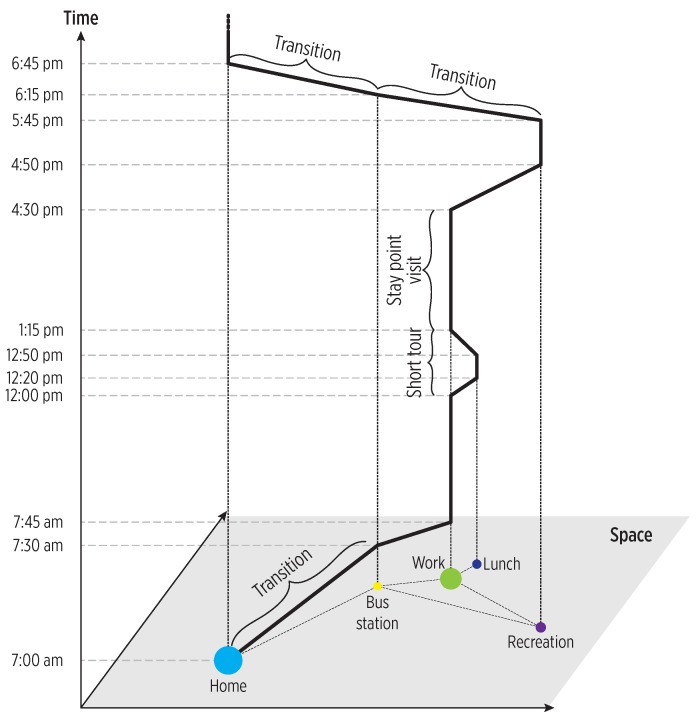
Conceptual representation of an individual’s mobility across space and time using the events related to POIs (enter, exit) as a reference. Adapted from [29]. Reprinted from Transportation Research Part C: Emerging Technologies, Vol 68, Chen, C.; Ma, J.; Susilo, Y.; Liu, Y.; Wang, M., The promises of big data and small data for travel behavior (aka human mobility) analysis, 285–299, Copyright (2016), with permission from Elsevier.

Detecting POIs from users and analyzing the POIs-users interaction through a large time scale can help to reveal a potential underlying spatio-temporal regularity. As shown in Figure 2, using the POIs as complex events, it is possible to study mobility in terms of visits and displacements between POIs. Particularly, we perform such study by involving the mobility events summarized in Table 3.

### 3.2. POIs Detection and Enter-Exit Events

We focus on detecting POIs and enter-exit events as the key to derive statistical properties for characterizing the dynamics of an individual’s mobility.

#### 3.2.1. Incremental POIs Computation

POIs are an average of observed locations, and their efficient on-the-go (on-line) calculation is of relevance in resource constrained IoT devices. Existing off-line algorithms require a complete trajectory to detect POIs [30]. Nevertheless, differential algorithms (Differential algorithms calculate the spatio-temporal differences between pairs of location fixes, rather than using a density-based or clustering approach), as the proposed in [21], are suitable for an incremental stream-based approach [25]. Using this idea, the pois_detector module in Figure 1 detects POIs (the $ev_{poi}$ event) as follows. The latitude and longitude coordinates of fixes under the $D_{max}$ and $T_{min}$ threshold parameters are accumulated (summation), rather than queuing fixes, which avoids growing memory requirements. If the spatio-temporal thresholds are met, a new POI’s centroid is simply calculated as the average of accumulated coordinates (see Equation (2)).

#### 3.2.2. Geofencing

A geofencing mechanism gf detects the enter-exit events from POIs using a voting-based scheme over a sliding window on the GPS stream. A simple thresholding of the latest fix proved not to be reliable during our experimental evaluation due to the variable accuracy of GPS receiver. The voting-based approach introduces some robustness to unreliable GPS fixes but also some latency. For low latency, it is crucial to keep a buffering limit.

**Figure 3:**
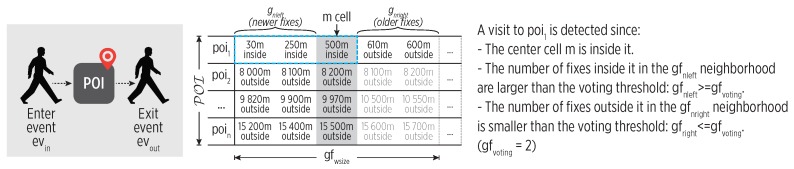
The Geofencing mechanism (gf). An enter to $poi_{1}$ is detected since we observe that newer (left) fixes are inside it and older fixes (right) are outside it.

Figure 3 illustrates the voting mechanism, which is described as follows. Let POI be the set of all POIs detected during a learning phase, $gf_{dist}$ a distance threshold, and *W* the sliding window of length $gf_{wsize}$ buffering the GPS stream. We split *W* into two neighborhoods around the center cell, $m=\left\lceil gf_{wsize}/2\right\rceil$. We count the distances between each fix and the centroids of all $POI_{i}\in \mathrm{POI}$ that are smaller than $gf_{dist}$ (i.e., inside the POI). $gf_{nleft}$ counts the distances for the left (newer) neighborhood, while $gf_{nright}$ counts the distances for the right (older) neighborhood. An enter event to a $POI_{i}$ is detected $ev(\mathrm{in},gf,\left\{POI_{i}\right\},t_{in})$ if the fix of the center cell is inside and if:

(3) $$gf_{nleft}\geq gf_{voting}\left(gf_{wsize}\right)\wedge gf_{nright}\leq gf_{voting}\left(gf_{wsize}\right)$$

$gf_{voting}$ depends upon the $gf_{wsize}$ and it defines the threshold of the voting scheme.

Similarly, an exit event from a POI is detected $ev(\mathrm{out},gf,\left\{POI_{i}\right\},t_{out})$ according to:

(4) $$gf_{nleft}\leq gf_{voting}\left(gf_{wsize}\right)\wedge gf_{nright}\geq gf_{voting}\left(gf_{wsize}\right)$$

### 3.3. Constructing and Updating the Cognitive Map

**Figure 4:**
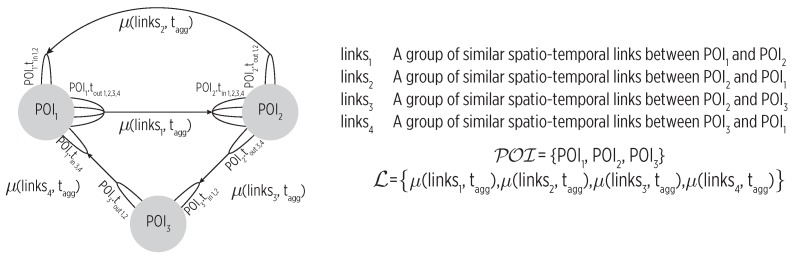
The cognitive map as a multigraph, where nodes represent the POIs and edges the time-annotated trajectories (links) followed by the user when moving between them.

The map_builder module constructs the cognitive map using the events detected by the perceptors in a bottom-up approach. This construction process attends to the spatial and temporal dependencies between events, which are of relevance to recognize complex human activities like mobility [27]. As shown in Figure 4, the map is a graph where nodes are POIs and edges are paths between them followed by the individual with typical transportation modes. The map is time annotated as we timestamp major events. Notice that the produced map will include nodes and edges that reflect the actual user’s mobility; in other words, the map construction process is not able to find regularity from inconsistent mobility, which will be reflected with many nodes and edges.

#### 3.3.1. Spatial Dimension

POIs detection undergoes a learning phase during some predefined time interval. At the beginning, the set of nodes in the model is empty, POI= {}. Once a new $POI_{det}$ is detected, we determine whether it should be added to the map as follows:

(5) $$\mathrm{classify}\left(POI_{det}\right)=\left\{\begin{array}{cc} \mathrm{visited} & \mathrm{if}(\overset{\left|\mathrm{POI}\right|}{\underset{i=1}{⋁}}S_{\mathrm{space}}\left(POI_{i},POI_{\det}\right)\leq \varepsilon_{d})\\ \mathrm{new} & \mathrm{else} \end{array}\right.$$

where ⋁ stands for the logical ***or*** operation, and $S_{\mathrm{space}}$ is a spatial similarity measure to determine the centroid equivalence of compared POIs. $POI_{det}$ is appended only if it is not spatially close (under $\varepsilon_{d}$ distance threshold) to any existing POI. After learning, POI holds the list of the *n* unique identified places, $\mathrm{POI}=\left\{POI_{1},POI_{2},\ldots ,POI_{n}\right\}$.

#### 3.3.2. Time Dimension

Whenever an $ev_{\mathrm{visit}}$ event to any POI is detected, the map_builder identifies a new spatio-temporal link expressed as $l_{\mathrm{new}}=link\left(POI_{\mathrm{out}},t_{\mathrm{out}},POI_{\mathrm{in}},t_{\mathrm{in}}\right)$ where $POI_{\mathrm{out}}\in \mathrm{POI}$ is the POI left at time $t_{\mathrm{out}}$, and $POI_{\mathrm{in}}\in \mathrm{POI}$ is the POI to which the moving entity entered at time $t_{\mathrm{in}}$.

We update the cognitive map as follows. We try to append the $l_{\mathrm{new}}$ link to one of the existing groups of similar spatio-temporal links, $L=\left\{links_{1},links_{2},\ldots ,links_{n}\right\}$. $l_{\mathrm{new}}$ is added to the group $links_{i}$, which must have the same origin and destination POIs, only if it turns out to be similar using:

(6) $$\mathrm{classify}\left(l_{\mathrm{new}}\right)=\left\{\begin{array}{ccc} \mathrm{similar} & \mathrm{if}\;(\overset{|links_{i}|}{\underset{j=1}{⋀}}S_{\mathrm{time}}\left(links_{i_{j}},l_{\mathrm{new}}\right) & \leq \varepsilon_{t})\\ \mathrm{different} & \mathrm{else} \end{array}\right.$$

where ⋀ stands for the logical **and** operation and $S_{\mathrm{time}}$ is a similarity measure for the enter and exit times of the compared spatio-temporal links within a threshold $\varepsilon_{t}$. If no similarity with existing link groups is found, then a new group $\left\{l_{\mathrm{new}}\right\}$ is appended to *L*.

Updating a $links_{i}$ group is followed by a $\mu (links_{i},t_{agg})$ digest (reduce) function to produce a single link that consolidates the enter and exit times of the group using the maximum, minimum, average, or another aggregate function $t_{agg}$. Thus, the actual edges of the cognitive map are $L=\left\{\mu \left(links_{1},t_{agg}\right),\mu \left(links_{2},t_{agg}\right),\ldots ,\mu \left(links_{n},t_{agg}\right)\right\}$.

## 4. Exploiting the Cognitive Map

Here, we describe how the PRM in a CDS architecture for on-device mobility detection can analyze, exploit and further refine the cognitive map information for purposes such as POIs relevance assessment and spatio-temporal mobility prediction. Notice that the quality of the outcome of these processes is highly dependent on the regularity described by the user, which is captured unaltered by the map construction process (Section 3).

### 4.1. POIs Relevance

The POIs relevance in daily mobility can be assessed through visit frequency and stay time information. Knowing the most relevant POIs for an individual provides a great opportunity for personalized services, not only mobility-related, but also in other domains. For instance, regarding health, the detection of only a few relevant places could represent sedentarism.To assess POIs relevance, let *P* be an observation period, e.g., a week, which is divided into equal subintervals $P_{k}$ such as days. Also, let $freq(POI_{i},P_{k})$ and $duration(POI_{i},P_{k})$ be the visits to and the stay time of $POI_{i}$ during time interval $P_{k}$, respectively. According to visits and stay duration, a POI can be graded as follows:

(7) $$W_{v}\left(POI_{i},P_{k}\right)=\frac{freq(POI_{i},P_{k})}{\sum_{j=1}^{\left|\mathrm{POI}\right|}freq(POI_{j},P_{k})}$$

(8) $$W_{t}\left(POI_{i},P_{k}\right)=\frac{duration(POI_{i},P_{k})}{\sum_{j=1}^{\left|\mathrm{POI}\right|}duration(POI_{j},P_{k})}$$

without loss of generality, a day is selected as the reference temporal scale.

We use $W_{v}$ and $W_{t}$ as features to categorize POIs into the set {mostly, occasionally, and exceptionally visited} as suggested in [17], and into the set {short, medium, and long stay time}, respectively, using k-means clustering. Other algorithms could be used; the key idea is that although users might share semantically equivalent POIs, their bounds (e.g., stay time) could differ, which calls for a clustering algorithm that self-adjusts to such bounds.

### 4.2. Mobility Estimation and Prediction

Mobility estimation infers the most likely POI at which a user is as a function of time:

(9) $$estimation\left(t\right)\to POI_{i}$$

where $POI_{i}\in \mathrm{POI}$ and *t* is the time or fragment of the day (morning, afternoon, night). Our cognitive map estimates a POI by traversing its nodes and selecting those link groups matching the specified *t*. As shown in Figure 4, multiple groups might be found but their probability is calculated as the transition frequency is also stored.

On the other hand, mobility prediction infers the next POI to be visited by a user:

(10) $$prediction\left(POI_{\mathrm{origin}}\right)\to POI_{\mathrm{destination}}$$

Although the efficiency of spatial-based HMM approaches for mobility prediction has been demonstrated [2,7], prediction requires relationships across time to be included [31]. Our cognitive map comprises both spatio-temporal attributes (edges L are time-annotated), helping to refine predictions using a timestamp or a look-ahead time window as follows:

(11) $$prediction\left(POI_{\mathrm{origin}},t\right)\to POI_{\mathrm{destination}}$$

Predictions are also generated by traversing the map graph. This is done using a breadth first search that filters out those link groups containing the desired $POI_{\mathrm{origin}}$ and *t* values. Predictions improve the smartphone mobility awareness, as it could infer the next POI and exit time upon detecting an $ev_{\mathrm{in}}$ event.

### 4.3. Refinement of the Cognitive Map Information

We also provide a preliminary mechanism to refine the information summarized in the cognitive map. Changes in mobility patterns (e.g., new POIs or different visit times and sequences) will cause the map to degrade, producing a large impact on the accuracy of estimations and predictions. A possible way to assess such degradation is the entropy of spatio-temporal inconsistences in mobility across time [2]. The entropy $H_{i}$ in the *i*th hour of the day can be calculated as:

(12) $$H_{i}=-\sum_{j=1}^{n}p_{i}\left(POI_{j}\right)\;log_{b}p_{i}\left(POI_{j}\right)$$

where *n* is the number of visited places and $p_{i}\left(POI_{j}\right)$ is the probability of user staying at $POI_{j}$, both during the *i*th hour. In turn, $p_{i}\left(POI_{j}\right)$ is calculated as:

(13) $$p_{i}\left(POI_{j}\right)=\frac{duration(POI_{j},p_{i})}{\sum_{k=1}^{\left|\mathrm{POI}\right|}duration\left(POI_{k},p_{i}\right)}$$

where $duration(POI_{j},p_{i})$ is the stay time at $POI_{j}$ and $\sum_{k=1}^{\left|\mathrm{POI}\right|}duration\left(POI_{k},p_{i}\right)$ is the total stay time at any POI during the *i*th hour. The entropy is low when the user holds consistent mobility patterns and high during inconsistent mobility patterns, e.g., during the learning phase. If the entropy is higher than a threshold, then the map could be updated by pruning the nodes (POIs) that are less visited and adjusting the temporal information of the link groups in L to ensure their reliability.

## 5. Evaluation and Experimental Results

We evaluated the performance of our cognitive map builder within the reference CDS considering the following aspects:

- Its mobility characterization features, including the spatio-temporal accuracy of events detection.
- Its ability for further on-device exploitation (mining).
- Its feasibility for on-device execution, including an energy consumption assessment.

We conducted short-term experiments on a rather small but fully annotated database, consisting on trajectory data from 5 users using a dedicated fast sampling (1 Hz) GPS logger (QStarz GPS Logger device, model BT-Q1000EX, Taipei, Taiwan). All users explicitly labeled the POIs in trajectories, which include regular Work and Home routines of several weeks. Overall, we collected 19 trajectories from 5 users (min 3.26 days, max 34.31 days, avg. 10.8 days). The mobility information of these trajectories refer to daily routines, such as Home-Work and Work-Home commutes during weekdays, as well as some leisure places during weekends. For all the trajectories, the participant users mostly employed a vehicle as a transportation mode for moving between rather distant places As this database is labeled, we could measure the accuracy of the basic events that are used to construct the map. We also explored the findings of our solution under inconsistent mobility; to do so, we employed a subset of the Cabspotting database [32], which includes mobility traces of taxi drivers, who are known for inherently irregular mobility; the subset was of 32 randomly selected 1-month taxi trajectories. Due to the many differences between our solution and existing works (no study of enter-exit events, no study of places categorization, etc.) we were unable to conduct a thorough comparison.

### 5.1. Implementation

We implemented the CDS reference framework with the map builder as a middleware for smartphones (Nexus 6 and Samsung Galaxy A5, with 3220 mAh and 2900 mAh batteries, respectively) running Android Operating System (OS) v7. We followed different workarounds to avoid energy-saving mechanisms at OS level that jeopardize continuous sampling [13]. We also implemented a desktop version of the CDS using the Python 3 language to quickly evaluate CDS functionality using different parameters. The desktop CDS version employs files with collected traces (latitude, longitude and timestamp) to construct the map, and it allows to configure the sampling rate to stress the system under different sampling conditions.The source code for the desktop version of our platform is available at https://github.com/s0lver/stm-creator.

It is worth noting that during its construction, the map evolves from a tensor to a directed multigraph. In the tensor, rows and columns hold the origin and destination POIs, so that when transition information from $POI_{i}$ to $POI_{j}$ is required, $cell_{i,j}$ would provide all the transitions between such places. Each $cell_{i,j}$ is in turn a container for each spatio-temporal link group $link_{i}$ of the list *L* from which we create the set of edges L.

### 5.2. Cognitive Map Construction

We launched our CDS with the parameters shown in Table 4. Note that these values are highly dependent on the information that shall be discovered (e.g., small values for $T_{min}$ and $D_{max}$ would reveal small POIs with short stay time). We focused on detecting relevant mobility attributes, hence the selected values.

**Figure 5:**
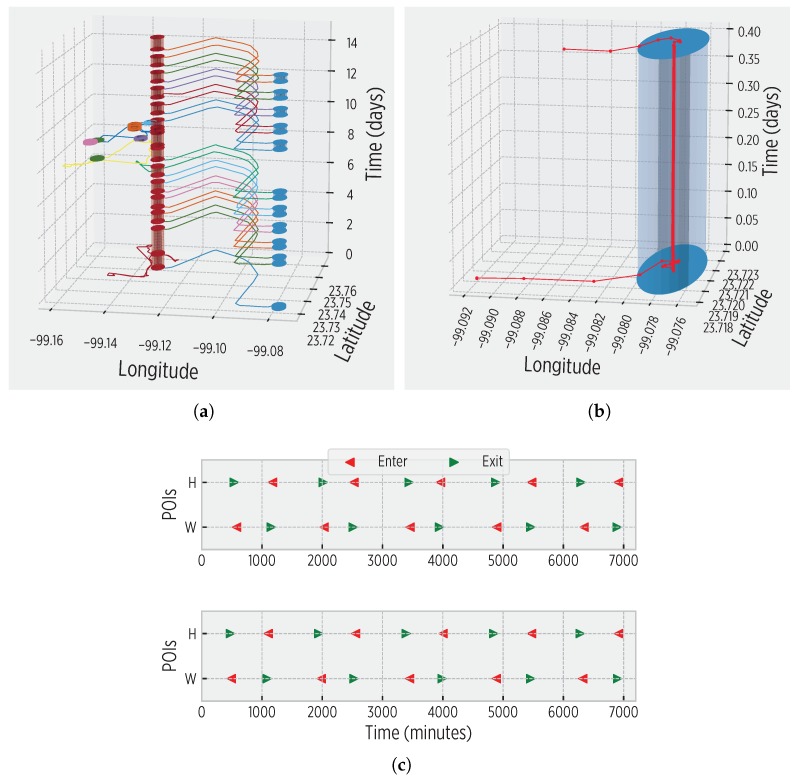
Spatio-temporal information autonomously derived by the system: (**a**) the cognitive map accounts for regularity, creating a summary of mobility represented in a tridimensional space; (**b**) a POI, represented by a cylinder whose height corresponds to the length of the visit; (**c**) a snapshot of enter-exit events to/from the Home (H, red) and Work (W, blue) POIs, which are dominant in typical user’s mobility.

Our CDS successfully created the cognitive map for all the trajectories of our annotated dataset. Figure 5 shows a 2-week snapshot of the mobility of user 1 autonomously captured by the smartphone. The detected POIs and enter-exit events demonstrate that the CDS effectively perceives the input information to construct the cognitive map. Note the regularity of user 1 in Figure 5; this information is promptly detected by the system to identify spatio-temporal mobility patterns that are incorporated in the cognitive map. The participant users validated the constructed cognitive maps using their labeled information.

Recall that the map construction process reflects the attributes of users mobility; as previously described, the cognitive map effectively detects patterns in regular mobility, but under inconsistent mobility there are no patterns to infer. In this regard, we also processed the Cabspotting database [32] to construct the corresponding maps. Our system identified a high irregularity caused by the inherent mobility of most taxi drivers. We present more insights about such irregularity later in this section.

### 5.3. Cognitive Map Characterization

We measured the spatio-temporal accuracy of the cognitive map in terms of the POIs centroid distance, time difference of enter-exit events, and missed visits with respect of ground truth data (1 Hz) and a varying sampling rate. We generated the ground truth data by processing our labeled dataset with the CDS. Then, we processed the trajectories using a base sampling rate (within the set {30,60,90,120,150,180} seconds) to study the degradation caused by reduced input data. This is relevant as IoT devices might not be able to collect location data with a high frequency due to energy constraints.

Table 5 shows the spatial accuracy of our map. Notice that despite the sampling rate, the missed POIs and centroid distance are small. Similarly, Table 6 shows the temporal accuracy of the events employed to construct the map. Again, although a slow sampling rate introduces some degradation in terms of missed visits and increased time differences, in overall the detected events offer a reasonable accuracy for map’s construction. Missed visits are short, as in our 1 Hz dataset their average length is no longer than 80 s regardless of sampling rate. Although the observed results are highly dependent on the employed sampling rate and user’s mobility, they provide evidence for the CDS to be able to characterize mobility.

### 5.4. Human Mobility Mining

We also evaluated the exploitation of the cognitive map’s information for human mobility mining tasks as follows.

#### 5.4.1. Place Categorization and POIs Relevance

**Figure 6:**
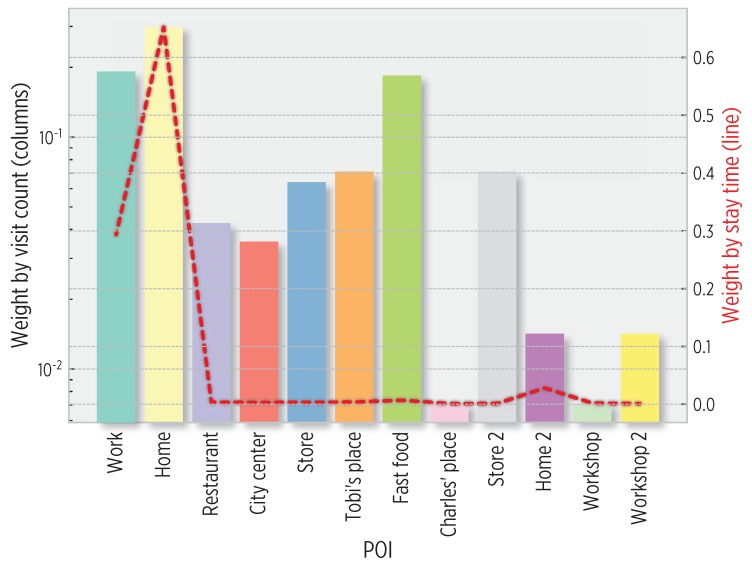
POI weights (%) based on visit count (Equation (7), logarithmic scale) and stay time (Equation (8)). Work and Home places have the largest stay time and visit count values, whereas Fast food is a frequently visited place with a small stay time.

We employed the stay time and visit frequency to POIs for their categorization using Equations (7) and (8). Figure 6 shows the weights calculated from the cognitive map for user 1. For this user with regular mobility, Home and Work places are the most important POIs in terms of visit count ($W_{v}$) and ($W_{t}$) stay time. Nevertheless, interesting attributes of other places could be inferred from data; for instance, the *Fast food* POI was occasionally visited but always during a short amount of time.

**Figure 7:**
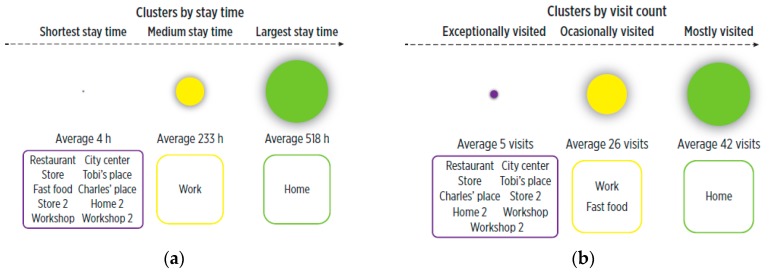
Categorization of the POIs according to temporal features: (**a**) stay time, (**b**) visit count.

By applying k-means clustering to the obtained weights, we categorized POIs by visit count (into mostly, occasionally and exceptionally visited places), and by stay time (short, medium and long stay time), setting k=3. Figure 7 shows both categorizations. Note that the centroids of these clusters (i.e., average stay times and visit counts) will be different for other users, as they interact with POIs at specific spatio-temporal scales. Since general thresholds are difficult to define, clustering techniques allow these groups to be user-tailored.

#### 5.4.2. Mobility Prediction

We also explored the potential of the constructed maps for mobility prediction using the simple traversal-based prediction mechanism discussed in Section 4.2. We used the fast sampling trajectories of our labeled dataset, defining one week as the learning period and the rest of each trajectory for testing. Focusing on the weekdays, after each exit event we generated a prediction for the next POI and enter time. As shown in Table 7, during these trials the simple traversal-based predictor achieved an accuracy around 53–91%, with time differences of 29–110 min. Again, note that since the map is constructed by strictly sticking to users mobility, a consistent mobility will produce a map with a reduced set of spatio-temporal links, which in turn will enhance predictions’ accuracy.

Furthermore, as stated in [2], given the diversity in prediction performance of different users, better prediction performance can be achieved for practical applications by applying different prediction models to users with distinct living habits. This is an issue that deserves to be further addressed in more detail, but the fact that the map includes a summarized representation of mobility represents a contribution toward more efficient prediction mechanisms that could be executed on-the-go.

#### 5.4.3. Entropy of Mobility

**Figure 8:**
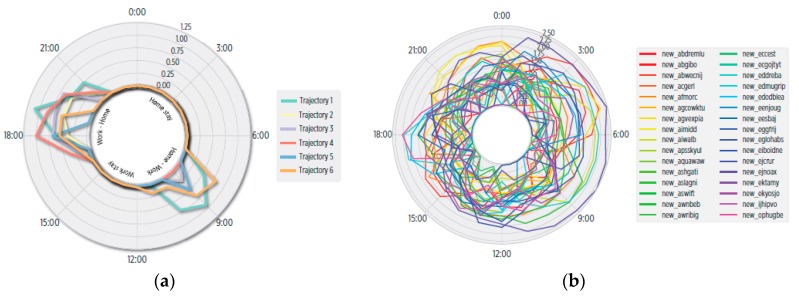
Hourly entropy in weekdays mobility. (**a**) For our user 1, small variations are found during 7–11 and 16–22, while in the rest of the day it is 0, as user described consistent mobility. (**b**) In the Cabspotting database, high entropy is found, highlighting the irregularity of participants’ mobility.

Finally, under mobility mining we also performed a preliminary study of the entropy in the individual’s mobility during weekday’s hours. As shown in Figure 8a, user 1 holds slight entropy variations during the intervals 7–11 and 16–22, which are explained by the differences of enter-exit times between *Home* and *Work* during mornings and evenings. The entropy is low for the rest of the day, which suggests consistent mobility. Evidently, under irregular mobility the entropy is expected to be higher. This was demonstrated after analyzing the Cabspotting database, on which we found high entropy values (Figure 8b) indicating the mobility inconsistence of participant taxi drivers.

Observing the entropy profile over longer intervals might help to identify when the information in the cognitive map is no longer valid. An increasing entropy might imply the update of the cognitive map to account for new mobility patterns. Nevertheless, such decision also depends on the mobility profile, as some people inherently have a high entropy, like taxi drivers, delivery carriers or salespeople, whose mobility is hard to predict as studied in [2]. We left for further study the definition of enhanced mechanisms based on the mobility entropy to specifically determine when the cognitive map can be safely updated.

### 5.5. Energy Overhead

Finally, using the following simple approaches, we measured the energy overhead caused by the map builder, which is of relevance in IoT systems with battery-powered devices.

#### 5.5.1. Energy Consumption with and without Cognitive Map

We measured the energy consumption of a fixed sampling rate under two conditions:

- The map builder was enabled (including events detection by gf and pois_detector).
- The map builder was disabled (GPS data was simply collected but unprocessed, i.e., the smartphone was mobility-agnostic).

To incorporate the consumption of accelerometer data processing, we also detected the transportation mode for each collected location fix. The transportation mode is useful to further refine the information in the map, for instance, to identify how users move between POIs. We ran experimental trials using the Nexus 6 smartphones, which were carried together by a user to observe the same mobility and GPS satellite signal conditions. We switched the role of each smartphone to reduce, at some extent, the battery health factor in the trials.

**Figure 9:**
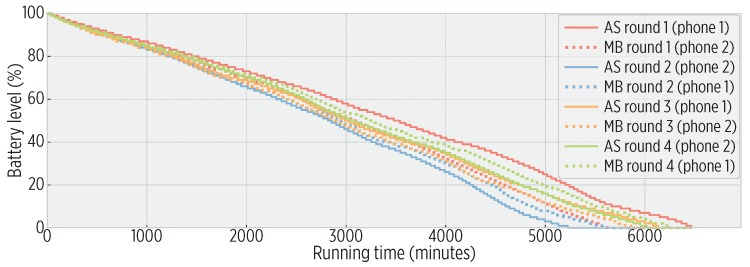
Energy overhead (seen as battery burnout) of the map builder with respect to an agnostic sampling rate. (AS = Agnostic Sampling system, MB = Map Builder system). In overall, the MB achieved 98% of AS running time, causing a small impact on energy.

Figure 9 shows the battery burnout of both implementations under a 30-second sampling rate. We also ran trials using a 60-second sampling rate. Overall, the smartphone executing the map builder achieved 98% of the running time of the agnostic smartphone, indicating only a slight impact on the energy resources of the mobile device.

#### 5.5.2. Energy Consumption of Perceptors

In the second approach, we estimated the energy overhead of perceptors using mobile apps that continuously invoked each perceptor using synthetic data (energy of data collection was discarded). We simulated an individual travelling around the world as the worst scenario for pois_detector and gf modules (i.e., continuously trying to create a POI, and never entering to any POI, respectively). For the gf module, we input 12 POIs to the cognitive map, causing 12 distance calculations per fix.

**Figure 10:**
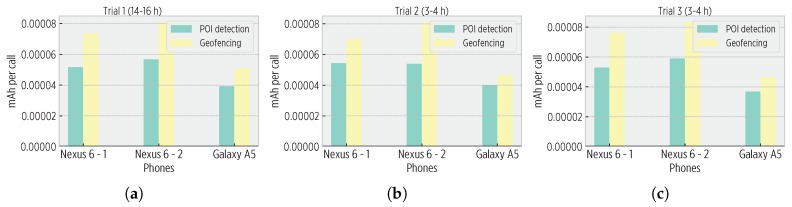
Energy overhead of perceptors different trials. (**a**) A trial of 14–16 h length, (**b**) A trial of 3–4 h length, (**c**) A trial of 3–4 h length. Based on the estimated consumption (mAh per call) and battery characteristics, the Nexus 6 would execute the pois_detector module for 60 h and the gf module for 46 h. the Galaxy A5 would achieve 20 h and 23 h, respectively.

Figure 10 shows the estimated energy consumption of perceptors in two different smartphones. Based on this data, the Nexus 6 could continuously execute the pois_detector and gf modules during 60.15 and 46.35 h, and the Galaxy A5, for 20.2 and 22.85 h. Notice that such continuous modules execution is not realistic, as energy would be consumed by other components and tasks run by the mobile platform. Nevertheless, from these results we do not identify a critical reduction of battery life; indeed, users are likely to charge their smartphones each day so that interrupted operation is avoided.

## 6. Discussion

The experimental results demonstrate the efficiency of the cognitive map to characterize user’s mobility with spatio-temporal accuracy with respect of high frequency GPS ground truth data. We also demonstrated that the cognitive map can be exploited for on-device mobility mining tasks such as POIs relevance, mobility prediction, and how its validity can be assessed via mobility entropy.

The cognitive map captures the regularity in user’s mobility (if any), which is relevant for further on-device mobility mining. In this regard, even a simple traversing-based prediction mechanism is possible thanks to the underlying graph of our map. Nevertheless, this could be improved with more complex strategies aimed at exploiting the spatio-temporal relationships for accuracy improvements. Moreover, additional experiments on mobility-diverse datasets are needed to obtain more conclusive assessments on prediction.

Although not explored in this article, mobility prediction can be exploited toward energy efficiency during POI visits and trajectories. In this regard, next POI predictions by the PRM might fail due to multiple factors causing uncertainty and risk. An active exploration approach aware of these factors could aid the system to update its predictions on the go. More specifically, mobility gradients [33] could account for the changes in distance when people move, selecting the POI with the lowest gradient as the predicted destination. Not every POI should be included in such calculation, only those with existing links to current POI. Also, fine-grain mobility from accelerometer could aid sampling rate adjustments based on the speed (transportation mode) of the user.

## 7. Conclusions

We proposed a spatio-temporal cognitive map focused on modeling mobility in a per-user basis. The construction of the map follows an event-based representation of mobility on which the events that arise from user interaction with POIs are accounted for spatio-temporal mobility characterization. Rather than isolated, the cognitive map is a fundamental component of a fully functional CDS implemented in smartphone devices, which has the advantage of being decentralized and following a personalized scheme that gradually constructs the cognitive map without off-line training. The construction process is simple and energy efficient, which is a very looked feature for restricted IoT devices. We also provided experimental evidence of the exploitation of the cognitive map, and presented the entropy of the observed mobility patterns as a possible way to assess the validity of the learned information. We stress that evaluation results will be highly dependent on the regularity of user’s mobility, which if exists will be reflected in the constructed map.

Moreover, we argue that CDS systems might allow IoT devices to adapt to their dynamic environment and adjust their performance parameters accordingly. Besides the interesting results obtained in this work, many issues concerning individuals’ mobility are of great interest for further research. Cognitive control should be explored taking advantage of the cognitive map to adaptively adjust GPS sampling rate during visits and trajectories, while preserving the information about individual’s mobility without much redundancy.

## Figures and Tables

**Table 1:** Table of symbols and notations.

Symbol	Description
*f*	A location fix
f.x,f.y,f.t	the latitude, longitude, and timestamp of a location fix *f*
pois_detector	the POIs detector module of our system
$T_{min}$	the minimum time threshold parameter for POIs detection
$D_{max}$	the maximum distance threshold parameter for POIs detection
$\left\{x_{poi},y_{poi}\right\},t_{in},t_{out}$	the centroid coordinates (x,y), and the enter and exit times of a detected POI
gf	the Geofencing module of our system
$gf_{dist}$	A distance threshold employed by the gf to determine if a fix is inside a POI
$gf_{wsize}$	the length of the Geofencing window
$gf_{nleft}$	the left neighborhood of (more recently collected) fixes
$gf_{nright}$	the right neighborhood of (older) fixes
$gf_{voting}$	the number of fixes required to consider a neighborhood to be inside a POI
map_builder	the map builder module of our system
POI	the set of POIs learned by the system
$\varepsilon_{d}$	A threshold to identify spatial equivalence between POIs
$link(POI_{out},t_{out,}$ $POI_{in},t_{in})$	A spatio-temporal link between two POIs and their transition times
$\varepsilon_{t}$	A threshold to identify temporal equivalence between POIs links
$links_{i}$	A group of spatio-temporal links
$t_{agg}$	An aggregation function for time (max, min, avg, etc.)
$\mu (links_{i},t_{agg})$	A function to summarize the temporal data of links in group $links_{i}$ using $t_{agg}$
*L*	the set of link groups before time summarization (μ)
L	the set of edges composing the cognitive map graph (after applying μ to *L*)
$freq(POI_{i},P_{k})$	the number of visits to $POI_{i}$ during the time interval $P_{k}$
$duration(POI_{i},P_{k})$	the stay time in $POI_{i}$ during time interval $P_{k}$
$W_{v}\left(POI_{i},P_{k}\right)$	the visit weight of $POI_{i}$ in user’s mobility during the time interval $P_{k}$
$W_{t}\left(POI_{i},P_{k}\right)$	the stay time weight of $POI_{i}$ in user’s mobility during the time interval $P_{k}$
$H_{i}$	the entropy in user’s mobility during the *i*th hour of the day
$p_{i}\left(POI_{j}\right)$	the probability of user staying at $POI_{j}$ during the *i*th hour of the day

**Table 2:** A comparison of the features in some proposed solutions for creating maps of human mobility.

	Suitable for on- Device Execution	Supports Irregular Location Sampling	Spatio-Temporal Links	Prediction Features	Privacy Issues
Off-line approaches [18,19,20,22]			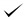	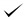	
Kang, et al. [23]	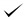	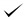			
Perez-Torres, et al. [25]	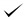	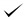			
This work	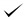	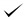	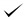	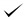	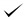

**Table 3:** Events of interest for mobility analysis based on POIs and their relationships.

id	Formal Definition	Description
$ev_{\mathrm{fix}}$	ev(fix,x,y,t)	A location fix provided by the GPS receiver.
$ev_{\mathrm{poi}}$	$ev(\mathrm{poi},pois\_detector,\left\{x_{poi},y_{poi}\right\},t_{in},t_{out})$	A POI detected by the pois_detector module.
$ev_{\mathrm{in}}$	$ev(\mathrm{in},gf,\left\{POI_{i}\right\},t_{in})$	A moving entity entered to $POI_{i}$, a relationship event.
$ev_{\mathrm{out}}$	$ev(\mathrm{out},gf,\left\{POI_{i}\right\},t_{out})$	A moving entity left $POI_{i}$, a relationship event.
$ev_{\mathrm{visit}}$	$ev(\mathrm{visit},map\_builder,\left\{POI_{i}\right\},t_{in},t_{out})$	A moving entity has visited $POI_{i}$, i.e., a sequence $s_{ev}=\left\{ev_{\mathrm{in}},ev_{\mathrm{out}}\right\}$ to $POI_{i}$ has been identified.

**Table 4:** Parameters set for the CDS and map builder.

Parameter	Value	Parameter	Value	Parameter	Value
$D_{max}$	500 m	$gf_{dist}$	250 m	$\varepsilon_{d}$	250 m
$T_{min}$	45 min	$gf_{wsize}$	3	$\varepsilon_{t}$	60 min

**Table 5:** Spatial accuracy of mobility events for constructing the cognitive map for different users and varying sampling rates. Variations in GPS accuracy and the lack of GPS signal indoors caused some POIs to be missed (M POIs = Accumulated missed POIs in trajectories for each user; C dist. = Average centroid distance).

Sampling Rate	Metric	User 1	User 2	User 3	User 4	User 5
		47 POIs	19 POIs	24 POIs	11 POIs	9 POIs
30	M POIs	0	0	0	0	0
	C dist.	3.12 m	36.09 m	9.29 m	19.04 m	11.86 m
60	M POIs	0	0	0	0	0
	C dist.	6.15 m	41.86 m	19.83 m	36.44 m	18.47 m
90	M POIs	0	0	0	0	0
	C dist.	10.86 m	66.88 m	38.84 m	55.61 m	17.90 m
120	M POIs	0	0	0	0	0
	C dist.	20.62 m	106.25 m	44.90 m	75.41 m	30.14 m
150	M POIs	0	0	1	0	0
	C dist.	24.19 m	175.01 m	78.84 m	93.09 m	30.73 m
180	M POIs	0	0	0	0	0
	C dist.	30.53 m	135.20 m	73.91 m	110.92 m	52.56 m

**Table 6:** Temporal accuracy of the mobility events to construct the cognitive map for different sampling rates. the slower the sampling rates, the larger temporal differences in mobility events. (MV = Accumulated missed visits in trajectories for each user; $t_{\mathrm{in}}$ diff. = avg. enter time difference, $t_{\mathrm{out}}$ diff. = avg. exit time difference).

Sampling Rate	Metric	User 1	User 2	User 3	User 4	User 5
		329 Visits	142 Visits	155 Visits	45 Visits	45 Visits
30	MV	20	3	2	0	2
	$t_{in}$ diff.	7.73 s	0.58 s	6.07 s	36.55 s	−111.85 s
	$t_{out}$ diff.	10.83 s	−4.88 s	−2.94 s	15.25 s	6.40 s
60	MV	46	10	5	2	7
	$t_{in}$ diff.	20.29 s	14.35 s	13.76 s	53.63 s	−125.31 s
	$t_{out}$ diff.	24.60 s	15.99 s	14.55 s	31.21 s	20.43 s
90	MV	56	14	11	2	10
	$t_{in}$ diff.	35.71 s	21.38 s	28.69 s	65.47 s	−86.61 s
	$t_{out}$ diff.	48.73 s	35.78 s	27.53 s	42.26 s	41.41 s
120	MV	52	18	14	3	10
	$t_{in}$ diff.	8.23 s	34.24 s	36.73 s	72.29 s	−95.67 s
	$t_{out}$ diff.	62.35 s	42.07 s	42.70 s	54.27 s	61.74 s
150	MV	57	22	15	5	10
	$t_{in}$ diff.	23.27 s	49.26 s	52.31 s	104.71 s	−82.41 s
	$t_{out}$ diff.	63.45 s	62.68 s	59.27 s	67.60 s	73.35 s
180	MV	53	31	17	7	10
	$t_{in}$ diff.	59.37 s	44.50 s	68.99 s	109.26 s	−95.32 s
	$t_{out}$ diff.	73.20 s	88.47 s	62.16 s	53.32 s	90.77 s

**Table 7:** Prediction accuracy of the simple traversing-based prediction approach.

User	Hits	Misses	Accuracy %	Average Time Difference
User 1	60	18	76%	29.7 min
User 2	7	6	53%	110.8 min
User 3	11	1	91%	33.9 min
